# Conclusiveness of the Cochrane Eye and Vision Group Reviews

**DOI:** 10.1186/s13104-015-1221-x

**Published:** 2015-06-16

**Authors:** Michael Mimouni, Francis Mimouni, Fani Segev

**Affiliations:** Department of Ophthalmology, Rambam Health Care Campus, P.O.B 9602, 31096 Haifa, Israel; Department of Pediatrics, Tel Aviv Medical Center, Tel Aviv and the Sackler School of Medicine, Tel Aviv University, Tel Aviv, Israel; Department of Ophthalmology, Meir Medical Center, Kfar Sava, Israel

**Keywords:** Cochrane, Ophthalmology, Eye and vision group, Reviews

## Abstract

**Background:**

To assess the conclusiveness of Cochrane Eye and Vision Group Reviews (EVGRs). We tested the hypotheses that: (1) the majority of EVGRs are inconclusive; (2) most reviews state the need for further and better studies; (3) the conclusiveness of a given review is affected by the number of randomized controlled trials (RCTs) included and the cumulative number of patients and eyes studied.

**Methods:**

A retrospective study of all EVGRs available in the Cochrane Library in June 2013. For each EVGR we recorded the number of RCTs found by the reviewers, the number of RCTs included for final analysis as fulfilling inclusion criteria, the total cumulative number of patients and eyes studied, the stated need for further and better studies, the reason stated for further studies and the type of conclusion reached by the reviewer(s). We used the Kruskal–Wallis test to determine differences between ‘‘conclusive’’ and ‘‘inconclusive’’ studies in terms of the outcome variables studied. The correlation between the number of included studies and cumulative sample size was studied using regression analysis.

**Results:**

Out of 106 EVGRs, 52.8% were conclusive. In 83.9% of the conclusive EVGRs one treatment/strategy/drug was found to be better than the alternative. The average number of available and included RCTs was significantly higher in conclusive EVGRs (P = 0.007 and P = 0.003 respectively). The total cumulative number of patients and number of eyes studied was approximately ten times higher in the conclusive EVGRs (P < 0.001 and P < 0.015 respectively). A similar percentage of RCTs was included in both conclusive and inconclusive reviews (76 vs. 73%). The vast majority of EVGRs, whether conclusive (84%) or inconclusive (96%), stated the need for further and better studies (P = 0.042). Fifty eight percent of the EVGRs justified the need for further studies for at least two reasons. The reason that was stated the most was a need for a larger amount of RCTs (67%).

**Conclusions:**

In approximately half of the cases, EVGRs allow the reader to reach a clinically applicable conclusion. Larger total cumulative participants, total cumulative number of eyes studied and number of RCTs performed all increase the likelihood of an EVGR to be conclusive.

## Background

In 1993, the Cochrane collaboration was founded in response to Archie Cochrane’s call for systematic reviews of randomized controlled trials (RCTs) in healthcare [1]. The collaboration is now a huge network of more than 28,000 volunteer contributors from over 100 countries [1].

The Cochrane collaboration has been noted by Grimshaw to be “the best single resource for methodologic research and for developing the science of meta epidemiology” [2]. The ultimate goal of the Cochrane reviews is to organize medical research information in a systematic way that allows end-users to reach practical conclusions in the interests of evidence based medicine [1].

It has been suggested that the goals of the Cochrane reviews are not always reached: indeed, not all Cochrane reviews are conclusive and even when they are it is often stated that there is a need for more and better studies [3–5]. Based on previous reports, 64–80% of Cochrane reviews are conclusive. However, a majority emphasizes the need for further studies [3–5]. Therefore, approximately one out of three times the reader is not able to reach a clinically applicable conclusion. To the best of our knowledge, no previous studies have reported on the conclusiveness of Cochrane reviews in the field of Ophthalmology.

We therefore designed the current study of all 106 Cochrane Eye and Vision Group Reviews (EVGRs) available until June 1 2013. We aimed to assess the conclusiveness of the EVGRs.

Similar to previous definitions we characterized conclusiveness by the ability or not to reach a practical definitive conclusion stating that one treatment was significantly better or not than another treatment or placebo [3–5]. Based upon our own previous experience\personal bias we hypothesized that: (1) the majority of the EVGRs are inconclusive; (2) most reviews state the need for further and better studies; (3) the conclusiveness of a given review is affected by the number of RCTs included in the review and the cumulative number of patients retained for analysis.

## Methods

We selected all of the 106 EVGRs available in the Cochrane Library on 1 June 2013 [6]. Two authors (MM and FM) independently extracted the following characteristics from each review: number of RCTs found by the reviewers on the topic, number of RCTs included for final analysis as fulfilling the criteria for inclusion, total cumulative number of patients enrolled in the included studies, total cumulative number of eyes studied (whenever available), stated need or not for further and better studies, reason stated for further studies and type of conclusion reached by the reviewers. A third reviewer was available if disagreement arose (FS). Many EVGRs report both the number of individuals recruited and the number of eyes studied and therefore the same principle was applied in this study. The reason stated for further study was prospectively classified into 7 subcategories: (1) need for further and better studies in a subgroup of patients in the review; (2) too small of a sample size in existing RCTs; (3) too small of a RCT number; (4) need for further studies to detect potential side effects; (5) need for further studies to determine long term outcomes; (6) need for further studies comparing multiple or new strategies; (7) other reasons.

The type of conclusion reached by the reviewers was classified into five subcategories: (1) the treatment/strategy/drug was found to be better than the alternative (other treatment/strategy/drug or placebo); (2) no significant differences were found between the two treatments/strategies/drugs; (3) no decision could be reached because the existing RCTs were of poor quality; (4) no decision could be reached because of not enough data; (5) no decision could be reached because of outdated existing RCTs. The first two categories of reviews were defined by us as ‘‘conclusive’’, and the last three as ‘‘inconclusive’’. For the purpose of determining conclusiveness, only the main outcome was considered.

Minitab version 16.1 (State College, PA, USA) was used for statistical analyses. We used the Kruskal–Wallis test to determine differences between ‘‘conclusive’’ and ‘‘inconclusive’’ studies in terms of the outcome variables studied. The correlation between the number of included studies and cumulative sample size was studied using regression analysis. Results are expressed as mean ± SD, median (range) or number (%). A *P* value of <0.05 was considered to be statistically significant.

## Results

We identified 106 EVGRs. The range, median, and mean number of RCTs examined, number of RCTs included for analysis, total cumulative number of patients enrolled, total cumulative number of eyes studied, the stated need or not for further and better studies and type of conclusion reached by the reviewers are described in Table 1. Briefly 56 (52.8%) EVGRs fulfilled our criteria for being conclusive versus 50 inconclusive (47.2%). Among the ‘‘conclusive’’ reviews, in 47 of them (83.9%) one treatment/strategy/drug was found to be better than the alternative and in 9 of them (16.1%) no significant differences were found between the two treatments/strategies/drugs. Among the EVGRs classified as ‘‘inconclusive’’ 5 (10%) were judged so because the RCTs were of poor quality, 44 (88%) because there were not enough data, and 1 (2%) because the RCTs were found to be outdated. The average number of available RCTs was significantly higher in conclusive EVGRs than in inconclusive ones (P = 0.007). The average number of included RCTs was significantly higher in conclusive reviews than in inconclusive ones (P = 0.003). Also, the total cumulative number of patients enrolled was more than ten times higher in the conclusive EVGRs than in the inconclusive ones (P < 0.001). In addition, the total cumulative number of eyes studied was nearly ten times higher in the conclusive EVGRs than in the inconclusive ones (P < 0.015). A similar percentage of RCTs was included in both conclusive and inconclusive reviews (76 vs. 73%). The vast majority of EVGRs, whether conclusive (84%) or inconclusive (96%), stated the need for further and better studies with the percentage being significantly higher in the inconclusive group (P = 0.042). The EVGRs justified the need for further studies as follows: 34% required further studies in subgroups, 17% required studies with larger sample sizes, 67% required a larger amount of studies, 12% required further studying of side effects, 29% needed monitoring of long term outcomes and 33% required further studies because of multiple or new strategies\drugs\therapies. Obviously, many of the reviews (58%), justified the need for further studies for at least two reasons.

**Table 1:** Study characteristics of the Cochrane Eye and Vision Group Reviews (EVGRs)

	‘‘Conclusive’’ EVGRs (n = 56)	‘‘Inconclusive’’ EVGRs (n = 50)	P
Number of RCTs^a^	10.7 ± 17.1	3.22 ± 5.57	0.003
Total cumulative number of patients enrolled^b,c^	984 (439–117,272)	50 (0–12,294)	<0.001
Total cumulative number of eyes studied^a,d^	1,295 ± 2,240	136.2 ± 287.9	0.015
Percent of EVGRs where further studies are needed^a^	96 ± 20	84 ± 37	0.042

*RCTs* randomized controlled trials, *EVGRs* Eye and Vision Group Reviews.

^a^Mean ± SD.

^b^Median (range).

^c^In 101 of the EVGRs the total cumulative number of participants could be retrieved, 52 of them were conclusive reviews and 49 of them were inconclusive ones.

^d^In 62 of the EVGRs the total cumulative number of eyes studies could be retrieved, 26 of them were conclusive reviews and 36 were inconclusive ones.

In a few of the reviews it was impossible to retrieve either the number of total cumulative participants (5%) and in many reviews it was impossible to retrieve the total cumulative number of eyes studied (42%) as they were not stated by the reviewers.

## Discussion

Contrary to our hypothesis, a very small majority of EVGRs was conclusive. In fact, 52.8% of EVGRs fulfilled our criteria for being conclusive. Thus, a researcher or clinician who wants a definite answer on a specific issue raised and summarized by the Cochrane Eye and Vision Group will get this answer approximately half of the time.

In the vast majority (83.9%) of the conclusive reviews one treatment\strategy or drug was found to be better than the alternative or placebo and only in 16.1% of conclusive reviews no significant differences were found. This point, is probably related to the fact that when a significant difference is found there is an intuitive conclusion that the answer is definitive, while when no differences are found (when the null hypothesis cannot be rejected) there is still a remaining issue of potentially insufficient power (that might still be improved by increasing the sample size). An alternative explanation could be publication bias. Indeed, articles with positive results are more likely to be published than those reporting negative results [7, 8]. They are also more likely to be accepted for publication in a journal of greater impact factor [9]. Thus, the total sample size of studies reporting positive results is likely to be larger than the total sample size of those reporting negative results. Indeed, from our study, it appears that the total number of available RCTs as well as the total number of retained RCTs, and subsequently the total number of participants and the total number of eyes studied were much higher in conclusive EVGRs than in inconclusive ones. The association between sample size and conclusiveness was also observed in previous reports in other fields of medicine [3, 4]. Interestingly, the percentage of RCTs included in the final analysis from all available RCTs was nearly identical between conclusive and inconclusive EVGRs. We speculate, that the stringent and systematic approach of the Cochrane Eye and Vision Group for including or excluding RCTs explains the consistency of exclusion percentages.

There was a stated need for further and better studies in 84% of conclusive EVGRs and in 96% of inconclusive ones. Although, the difference between 96 and 84% was statistically significant (P = 0.042), it is still apparent that in both groups the vast majority of EVGRs stated such a need. Fifty eight percent of the EVGRs justified the need for further studies for at least two reasons but the reason that was stated the most was a need for a larger amount of studies (67%). The second most frequently stated reason was the need for further studies in subgroups (34%), while the third most frequently stated reason was the need for further studies because of multiple or new strategies\drugs\therapies (33%). Thus, it appears that in most cases whether or not current science supports one type of evidence, the Cochrane Eye and Vision Group often concludes that there is room for more evidence, although there are limited resources for research.

It is worth noting that three of our previous reports of the conclusiveness of Cochrane reviews in neonatology [3], pediatric-gastroenterology [4] and nutrition [5] demonstrated higher rates of conclusive reviews (67.7, 80 and 64.4% respectively) when compared to that of the EVGRs. On one hand, this perhaps provides us with a state of play on how much of the ophthalmic practice is well supported by high-level evidence when compared to the previously mentioned fields despite the upward growing trend of overall publications in the field of ophthalmology [10]. On the other hand this may be the result of the Eye and Vision Group dealing with novel and “problematic” clinical questions which have yet to be investigated sufficiently.

A limitation of the EVGRs was that it was not always possible to retrieve either the number of total cumulative participants or the total cumulative number of eyes studied as they were not stated by the reviewers. We suggest that there is room for improvement in the reporting of RCTs which should all clearly state both numbers. Another limitation of the study is that other characteristics of EVGRs such as the types of comparisons included and how broad or narrow the questions are were not collected and analyzed. In addition, the field of Ophthalmology is so highly specialized and therefore, retina specialists, for instance, may have better success with using EVGRs to answer clinical questions as opposed to neuro-ophthalmologists or glaucoma specialists. An additional limitation of this study is that though it does not provide insight regarding the quality of the EVGRs or their usefulness. An inconclusive review may be of very good quality and be thorough, of great help to the end-user (summarizing evidence from many sources into one article), and pertinently identifying gaps in our understanding and priorities for future research. In fact, an inconclusive review may be more useful than a conclusive one. Finally, the primary purpose of Cochrane reviews is really to establish a level of evidence by performing a systematic review of the literature. Its primary purpose might not be to “render decisions” regarding whether a treatment is better or not, and in fact it may appear that Cochrane review groups discourage authors from being prescriptive in their discussion and conclusions. In addition, Cochrane reviews provide information on other aspects of treatment such as side-effects encountered but the scope of our study was to review the primary outcomes of the reviews.

## Conclusions

In summary, we found that the EVGRs allow, in approximately half of the cases, the reader to reach a clinically applicable conclusion. The Cochrane Eye and Vision Group, in a large majority of the reviews, emphasize the weaknesses of both conclusive and inconclusive reviews and suggest avenues for future and better research. The percentage of conclusive Cochrane reviews is lower in ophthalmology than in other fields of medicine studied in a similar fashion.

## Abbreviations

RCTs: randomized controlled trials
EVGRs: Cochrane Eye and Vision Group Reviews
